# Occupational Difference in Association of Poor Sleep Quality and Metabolic Syndrome: Differences between Workers and Employees

**DOI:** 10.1155/2021/9947027

**Published:** 2021-09-17

**Authors:** Sima Hashemipour, Zohreh Yazdi, Azam Ghorbani

**Affiliations:** Metabolic Diseases Research Center, Research Institute for Prevention of Non-Communicable Diseases, Qazvin University of Medical Sciences, Qazvin, Iran

## Abstract

**Background:**

Regarding insufficient data about interaction of job in association of sleep quality with metabolic syndrome (MS), this study has been designed to evaluate this association in workers and employees.

**Methods:**

This cross-sectional study was conducted on 448 municipal staff (employee group: *N* = 295; worker group: *N* = 153) referring for periodic examinations. The relationship between sleep quality and MS and their relevant components was investigated in both groups.

**Results:**

In the worker group, poor sleep quality was independently associated with the risk of MS by 3.04 times (*P* < 0.01). Among the components of metabolic syndrome, hypertriglyceridemia was associated with a greater number of sleep disorder components. There was no association between metabolic syndrome and sleep quality in the employee group.

**Conclusion:**

Poor sleep quality exerts different effects on metabolic complications in employees and workers.

## 1. Introduction

Metabolic syndrome refers to a cluster of cardiovascular risk factors such as abdominal obesity, hypertension, and impaired plasma lipid or glucose [1]. The prevalence of metabolic syndrome is high, and about one-third of the Iranian general population suffers from this syndrome [1]. Since this syndrome is associated with a number of complications including cardiovascular diseases, diabetes, fatty liver, and even some cancers [2], investigating its contributing factors is of particular importance. Several factors are involved in the development of metabolic syndrome, some of which include genetic factors, poor nutrition, and physical inactivity [3]. In recent years, the role of factors such as psychological factors [4] and sleep disorders in the development of this syndrome has also been addressed.

Recent studies have demonstrated that sleeping plays a more important role in metabolic health and is not just some physical resting. Sleeping process is associated with major changes in the hormones involved in metabolism, such as cortisol and growth hormone as well as various neuropeptides [5]. During wakefulness, metabolism is associated with increased levels of free radicals; in contrast, when the metabolic rate and body temperature are reduced, especially when the brain is in the non-REM sleep stage, there is an opportunity to repair the damage caused by free radicals [6]. Sleep disorders can also lead to excessive activity of the sympathetic nervous system, which can have potential effects on complications such as hypertension [7].

Although several pathological mechanisms have been identified for the role of sleeping in metabolic health, the results of clinical and epidemiological studies on the association between sleep disorders and metabolic syndrome are quite inconsistent. In some studies, metabolic disorders have only been associated with sleep duration [8], while in some others, they have been correlated with overall sleep quality and its components [9, 10]. Some studies have also reported no association between sleep duration and quality and metabolic complications [11, 12].

Given that most of these studies have used similar measurement, i.e., Pittsburgh Sleep Quality Index (PSQI) and statistical methods to assess sleep quality, it seems that their inconsistent results are mostly due to the differences in the studied population.

Some evidence has been reported on gender and ethnic differences in metabolic or inflammatory responses to sleep disorders [11, 13]. However, the vast majority of studies have been focused on the association between different components of sleeping and metabolic complications in the general population or a particular group, and few studies have compared different groups in this regard. In addition, there is some evidence that socioeconomic status can increase metabolic complications [14]. Our main question was “Is there any difference in the association of sleep quality with metabolic syndrome between two highly different characteristics (such as income) of occupations?”.

To answer this question, the association between sleep quality and metabolic syndrome was compared between two groups working in the municipality, including office employees and workers.

## 2. Materials and Methods

### 2.1. Study Population

This cross-sectional study was performed on staff working in XXX (blinded by request from JOEM) Municipality in XXX (blinded by request from JOEM) who referred for annual occupational examinations. The study was approved by the ethics committee of XXX (blinded by request from JOEM) University of Medical Sciences (approval code: XXX (blinded by request from JOEM)). REC.1396.47 and the principles outlined in the Declaration of Helsinki have been followed. Including criteria were age ≥ 20 years old and working as a worker or an employee; excluding criteria were any acute disease in recent one month and chronic kidney or liver diseases (except fatty liver) or history of cancers in recent one year. Based on these criteria, 448 staff (153 workers and 295 employees) included a questionnaire including participants' demographic characteristics, occupation type, shift work, family history, medical history, and drug abuse that was completed.

### 2.2. Anthropometric Assessment

The participants' weight was measured and recorded with minimum clothing on and without wearing shoes using a digital scale with the accuracy of 100 g. Their height was measured using a tape measure in a standing position next to the wall and without wearing shoes, while their shoulders were in normal conditions with the accuracy of 1 cm. Body mass index (BMI) was calculated as weight (in kg) divided by the square of height (in m^2^). The waist circumference was measured at its narrowest part after one normal exhalation using a nonelastic tape measure without imposing any pressure on the person's body with the accuracy of 0.5 cm. Arterial blood pressure was measured by the physician in the right arm of the person after 15 min resting by a standard Welch Allyn mercury sphygmomanometer with the accuracy of 5 mm Hg. It was measured twice, and the mean value was recorded as the person's arterial blood pressure.

### 2.3. Biochemical Evaluation

After 12 to 14 hours of fasting, the venous blood samples were taken to measure blood glucose and serum lipid levels (including blood triglycerides, total cholesterol, and HDL-c). On the same day, the blood glucose was measured using the colorimetric glucose oxidase method. Serum blood samples were then centrifuged at 3000 rpm for 10 min and kept at the negative serum temperature of 70°C. Serum HDL-c levels were assessed after the precipitation of apoB-containing lipoproteins using phosphotungstic acid solution.

### 2.4. Diagnostic Evaluation of Metabolic Syndrome and Sleep Quality

According to the ATP III criteria, metabolic syndrome was defined as having three or more of the following risk factors: waist circumference greater than 88 cm in females and more than 102 cm in males, serum TG level greater than 150 mg/dl, serum HDL cholesterol level less than 50 mg/dl in females and less than 40 mg/dl in males, systolic blood pressure greater than 130 mmHg or diastolic blood pressure greater than 85 mmHg, taking antihypertensive medications, and fasting blood sugar level greater than 100 mg/dl [1].

Participants' sleep quality was assessed by self-expression using PSQI. This scale consists of 19 items, measuring seven components of sleep quality, including sleep latency, sleep disturbances, subjective sleep quality, daytime dysfunction, sleep duration, using sleep medications, and habitual efficiency. In each above item, higher scores indicate worse quality of that component. The overall score is obtained from the sum of all items. A higher PSQI score indicates worse sleep quality. A PSQI score above 5 is defined as poor sleep quality [15].

The data were recorded in the research questionnaires after the interview. Researchers interviewing and evaluating the anthropometric indicators were unaware of the biochemical test results. The ambiguous data were resolved and clarified by making phone calls to the participants.

### 2.5. Statistical Analysis

The independent *t*-test and chi-squared test were used, respectively, to compare the quantitative and qualitative data. To investigate the independent association between sleep quality and its components and metabolic syndrome, the logistic regression was performed by adjusting age, gender, BMI, level of education, and shift work. A *P* value less than 0.05 was considered as the significance level. Data analysis was performed using SPSS software (ver. 20.0).

## 3. Results

Results were obtained from 448 people (295 employees and 153 workers). Demographic findings, frequency of metabolic syndrome and its components, and overall quality of sleep and its components are compared in Table 1.

The frequency of female gender, high level of education, and hypertriglyceridemia were higher in the employee group, while hypertension was more prevalent in the worker group. There were no statistically significant differences in the mean score of sleep quality, frequency of poor sleep quality, and frequency of metabolic syndrome between the two groups. The mean daytime dysfunction and sleep duration were higher in the employee group, while the mean subjective sleep quality was higher in the worker group.

The associations between the components of metabolic syndrome and sleep quality in the worker and employee groups are shown in Tables 2 and 3, respectively. In the worker group, the sleep quality and most of its components (including subjective sleep quality, sleep latency, sleep disturbances, and daytime dysfunction) were significantly worse in the case of metabolic syndrome (*P* < 0.01 for sleep quality, sleep disturbances, and subjective sleep quality; *P* < 0.05 for sleep latency and daytime dysfunction).

In workers, among metabolic syndrome components, hypertriglyceridemia was more associated with impaired sleep components (worse sleep quality, habitual sleep efficiency, and subjective sleep quality).

Also, in workers with abdominal obesity, the mean subjective sleep quality and sleep disturbances were worse compared with workers with normal waist circumference. Impaired fasting sugar and low HDL level were accompanied with higher sleep disturbances score in workers.

In employees, sleep quality and its components were not significantly different in subjects with and without metabolic syndrome (Table 3). Among metabolic syndrome components, only low HDL and abdominal obesity were accompanied with higher sleep disturbances score, and habitual sleep efficiency was worse in the hypertriglyceridemia group (all of them *P* < 0.05).

Results of logistic regression analysis for association of sleep quality (as independent variable) with metabolic syndrome (as dependent variable) are shown in Table 4. The risk of metabolic syndrome increased up to 2.76 times among workers with poor sleep quality (*P* < 0.01). After adjusting the age, gender, shift work, BMI, and level of education, this increased risk still remained significant (odds ratio = 3.04, *P* < 0.05).

The amount of increased risk of metabolic syndrome with each unit worsening of sleep quality components also is shown in Table 4, so that as the subjective sleep quality was deteriorated by one score, the adjusted risk of metabolic syndrome increased by 2.2 times in workers (*P* < 0.01). Also, for every score of worsening sleep disturbance or sleep latency, the adjusted risk of metabolic syndrome increased about 2.4 times in workers (*P* < 0.05 and *P* < 0.01, respectively). As the daytime dysfunction worsened by one score, the risk of metabolic syndrome increased about twice in workers (*P* < 0.01). In employees, poor sleep quality or worsening its components was not associated with increased risk of metabolic syndrome before and after adjustment (Table 4).

## 4. Discussion

In this study, the association between sleep quality and metabolic syndrome was significantly affected by the type of occupation. Although there was no significant difference in the frequency of metabolic syndrome and total sleep quality score between the two groups, the sleep quality and its components were associated with metabolic syndrome only in the worker group, so that workers with poor sleep quality were about three times more likely to develop the metabolic syndrome, even after adjusting other risk factors for this syndrome. In contrast, no association between poor sleep quality with metabolic syndrome was found in the employee group. In recent years, various studies have been conducted on the association between metabolic syndrome and sleep quality; the results of which are quite inconsistent.

According to the meta-analysis conducted by Lian et al., 16 eligible studies were included to investigate the association between overall sleep quality and metabolic syndrome. Although in the final meta-analysis, the overall poor sleep quality was associated with the 1.37% risk of developing metabolic syndrome; in most of the included studies, the association between sleep quality and metabolic syndrome was not significant despite their good power [16]. The researchers concluded that some unknown factors have affected the heterogeneity of the results.

Zohal et al. conducted a study on 1079 people among the Iranian general population and found no significant difference in the overall sleep quality between the metabolic syndrome group and the normal group. However, some components of sleep quality such as the use of sleeping medications and sleep disturbances were higher in the group with metabolic syndrome [17]. Also, in some studies conducted on specific populations, no association was observed between sleep quality and metabolic syndrome. For example, in the study of Chang et al. conducted on Taiwanese police officers [18], in the study of Yoo and Franke on the US law enforcement officers [8], in the study of Kazman et al. (2012) on African-American people [11], and in the study of Lajoie et al. on Canadian rotating shift nurses [12], no association has been found between the overall sleep quality and metabolic syndrome.

In contrast, in some other studies, poor sleep quality has been associated with the increased risk of metabolic syndrome. The study by Jennings et al. was one of the first studies which examined the association between metabolic syndrome and sleep quality among 210 volunteers aging 30 to 45 years old of general population. In this study, the metabolic syndrome risk increased by 1.4 times for each SD increase in sleep quality score (representing the worsening of sleep quality) [19]. In the study of Hung et al. on the Taiwanese general population [9], the study of Lee et al. on Korean adults referring to the university-affiliated hospital clinic [20], and the study of Okubo et al. on the Japanese general population [10], the association between sleep quality and metabolic syndrome has been reported. In these studies, the metabolic syndrome risk in groups with poor sleep quality was 2-4 times higher than that in people with good sleep quality.

In addition to the association between overall sleep quality score and metabolic complications, it is also important to examine how sleep quality components are associated with this syndrome.

In the present study, worsening of sleep disturbances, subjective sleep quality, sleep latency, and daytime dysfunction were associated with increased risk of metabolic syndrome in workers. After adjusting for the other risk factors of metabolic syndrome, the association of all these components with this syndrome still remained significant.

According to other studies, the association between some components of sleep quality, such as sleep latency and frequent sleep disturbances with metabolic complications, seems to be more consistent. In the study conducted by Okubo et al., scores of 2 and 5 on sleep latency were associated with 3.8 and 5.9 times of the risk of metabolic syndrome, respectively (compared to the score of zero). Scores of 1 and 2 on sleep disturbances were also associated with 2.4 and 3.8 times of the risk of metabolic syndrome, respectively (compared to the score of zero) [10]. In the meta-analysis performed by Lian et al., among the components of sleep quality, sleep latency and difficulty in remaining asleep were associated with the increased risk of metabolic syndrome [16].

One of the most important pathophysiological etiologies of metabolic complications of impaired sleep quality, particularly sleep latency and sleep disturbances, is increased hypothalamic-pituitary-adrenal axis (HPA axis) and sympathetic system activities. In the study by Stamatakis and Punjabi, development of sleep fragmentation using auditory or mechanical stimuli in healthy participants for two nights was associated with decreased insulin sensitivity and clearance of insulin-independent glucose. The cortisol level and sympathetic system activity increased in these people on the day after the test [21].

Elevated sympathetic nervous system activity and cortisol level increase gene expression for the production of inflammatory factors. Increased cortisol and adrenergic counter-regulatory hormones as well as persistent mild inflammatory conditions led to metabolic complications such as increased insulin resistance and metabolic syndrome [22]. However, there is a bidirectional association between cytokines and sleep, and the increasing cytokines such as interleukin-6 can induce fatigue and poor sleep quality [23].

The main finding of our study was that the different associations of sleep quality and metabolic syndrome between the worker and employee groups. Unfortunately, enough studies have not been conducted to compare different social and occupational groups and investigate the factors affecting the association between sleep quality and metabolic complications. However, there are indirect evidences for producing some hypotheses in this regard.

In the study of Jennings et al., sleep quality was associated with insulin resistance, metabolic syndrome, and its components (waist circumference and plasma glucose). However, after adjustment of depression, there was no association between sleep quality and metabolic syndrome [19].

In the study by Nguyen-Rodriguez et al., high sleep latency was associated with emotional eating. The results of multivariate models showed that trait anxiety was an essential factor for emotional eating in people with high sleep latency [24].

Therefore, it seems that psychological factors also play an important role as mediators in the association between sleep quality and metabolic complications. In addition, socioeconomic factors are effective in the development of metabolic complications. In recent years, despite the improvement in cardiovascular risk factors in the general population, there has been an increase in some cardiovascular risk factors, such as the prevalence of diabetes in groups with low socioeconomic status [25].

Our study had strengths as well as some limitations. One of the limitations of our study was the lack of psychological and socioeconomic measurement and adjustment of findings with these factors. Another limitation was the cross-sectional design of the study, which limited the identification of cause-and-effect in the two variables of sleep quality and metabolic complications. The strength of our study was in the investigation of the relationship between sleep quality and metabolic syndrome in two separate occupational groups with significant social differences.

## 5. Conclusion

In short, in our study, the association between sleep quality and metabolic syndrome and its components was only observed among the workers. In future investigations, it is necessary to examine intermediate factors such as psychologic and socioeconomic factors that can affect the relationship between sleep quality and metabolic complications.

## Figures and Tables

**Table 1:** Comparison demographic data, metabolic syndrome components, and sleep quality components between groups of workers and employees.

Variable	Workers (153)	Employees (295)	*P* value
Age	37.7 ± 8.0	38.4 ± 6.5	NS
Gender (percent of females)	2.6%	24.3%	<0.0001
Education			
No formal education	20.1%	0.7%	<0.0001
Middle or high school	20.8%	1.0%	
Diploma or associate′s degree	40.9%	23.6%	
Bachelor or higher	18.2%	74.7%	
Marriage status (percent of married)	90.3%	90.9%	NS
Shift work	35.1%	2.7%	<0.0001
Smoking	12.7%	8.9%	NS
Hypertension	46.1%	29.7%	<0.001
Hypertriglyceridemia	32.5%	45.6%	<0.01
Low HDL	64.9%	70.9%	NS
IFG	23.4%	18.6%	NS
Abdominal obesity	22.1%	26.0%	NS
Metabolic syndrome	29.2%	30.4%	NS
Number of metabolic syndrome components	1.88 ± 1.27	1.9 ± 1.25	NS
Subjective sleep quality	0.83 ± 0.72	0.69 ± 0.61	<0.05
Sleep latency	0.71 ± 0.75	0.66 ± 0.70	NS
Sleep duration	1.03 ± 0.84	1.22 ± 0.78	<0.05
Habitual sleep efficiency	0.85 ± 0.41	0.74 ± 0.39	NS
Sleep disturbances	0.99 ± 0.56	0.94 ± 0.47	NS
Daytime dysfunction	0.82 ± 0.77	1.01 ± 0.76	<0.05
Using sleep medications	0.13 ± 0.58	0.09 ± 0.46	NS
PSQI	4.6 ± 2.33	4.71 ± 2.24	NS
Poor sleep quality	30.7%	0.31.2%	NS

IFG: impaired fasting glucose; PSQI: Pittsburgh Sleep Quality Index. Abdominal obesity: according to ATP III criteria: waist circumference ≥ 88 cm in females and ≥102 cm in males. Poor sleep quality: PSQI ≥ 5.

**Table 2:** Association of sleep quality components with metabolic syndrome and its components in the worker group.

	Metabolic syndrome		Hypertriglyceridemia		Low HDL		IFG		HTN		Abdominal obesity	
	+(45)	-(108)	+(50)	-(103)	+(99)	-(54)	+(35)	-(118)	+(71)	-(82)	+(34)	-(119)
Sleep quality	5.49 ± 2.15^∗∗^	4.24 ± 2.31	5.16 ± 2.13^∗^	4.33 ± 2.38	4.86 ± 2.09	4.12 ± 2.66	4.86 ± 2.09	4.13 ± 2.66	4.57 ± 2.32	4.63 ± 2.35	5.15 ± 2.32	4.45 ± 2.32
Subjective sleep quality	1.06 ± 0.80^∗∗^	0.73 ± 0.66	1.00 ± 0.69^∗^	0.74 ± 0.72	0.88 ± 0.66	0.72 ± 0.81	0.88 ± 0.66	0.72 ± 0.81	0.77 ± 0.72	0.87 ± 0.72	1.06 ± 0.74^∗^	0.76 ± 0.71
Sleep latency	0.91 ± 0.85^∗^	0.63 ± 0.70	0.80 ± 0.80	0.66 ± 0.73	0.76 ± 0.76	0.61 ± 0.73	0.76 ± 0.76	0.61 ± 0.73	0.74 ± 0.78	0.68 ± 0.73	0.79 ± 0.91	0.69 ± 0.71
Sleep disturbances	1.2 ± 0.66^∗∗^	0.90 ± 0.50	1.10 ± 0.54	0.94 ± 0.57	1.06 ± 0.55^∗^	0.87 ± 0.58	1.06 ± 0.54^∗^	0.87 ± 0.58	1.04 ± 0.62	0.97 ± 0.52	1.18 ± 0.52^∗^	0.94 ± 0.57
Daytime dysfunction	1.04 ± 0.79^∗^	0.73 ± 0.76	0.98 ± 0.79	0.74 ± 0.76	0.85 ± 0.70	0.76 ± 0.91	0.85 ± 0.70	0.75 ± 0.90	0.81 ± 0.78	0.82 ± 0.78	0.88 ± 0.73	0.81 ± 0.79
Habitual sleep efficiency	0.04 ± 0.21	0.1 ± 0.47	0.20 ± 0.67^∗^	0.03 ± 0.17	0.10 ± 0.48	0.05 ± 0.23	0.10 ± 0.48	0.05 ± 0.23	0.04 ± 0.20	0.12 ± 0.53	0.05 ± 0.24	0.09 ± 0.45
Sleep duration	1.08 ± 0.84	1.00 ± 0.85	0.96 ± 0.85	1.06 ± 0.84	1.06 ± 0.84	0.98 ± 0.86	1.05 ± 0.85	1.02 ± 0.85	1.07 ± 0.86	1.00 ± 0.83	1.05 ± 0.88	1.02 ± 0.84
Use of sleep medications	0.13 ± 0.54	0.13 ± 0.53	0.12 ± 0.52	0.14 ± 0.54	0.13 ± 0.48	0.14 ± 0.62	0	0.17 ± 0.60	0.11 ± 0.42	0.15 ± 0.61	0.11 ± 0.53	0.14 ± 0.54

Data are presented as the mean ± SD. Comparison of sleep quality components between subjects with and without metabolic risk factors was performed using *t*-test. ^∗^*P* < 0.05 and ^∗∗^*P* < 0.01.

**Table 3:** Association of sleep quality components with metabolic syndrome and its components in the employee group.

	Metabolic syndrome		Hypertriglyceridemia		Low HDL		IFG		HTN		Abdominal obesity	
	+(89)	-(206)	+(134)	-(161)	+(209)	-(86)	+(55)	-(240)	+(87)	-(208)	+(77)	-(218)
Sleep quality	4.84 ± 2.29	4.66 ± 2.22	4.84 ± 2.29	4.60 ± 2.20	4.86 ± 2.19	4.34 ± 2.32	4.61 ± 2.49	4.73 ± 2.18	4.67 ± 2.32	4.73 ± 2.21	4.96 ± 2.13	4.62 ± 2.28
Subjective sleep quality	0.67 ± 0.61	0.70 ± 0.62	0.69 ± 0.63	0.69 ± 0.60	69 ± 0.62	0.68 ± 0.59	0.69 ± 0.60	0.69 ± 0.62	0.70 ± 0.59	0.69 ± 0.63	0.67 ± 0.57	0.70 ± 0.63
Sleep latency	0.68 ± 0.73	0.66 ± 0.69	0.66 ± 0.71	0.67 ± 0.69	0.70 ± 0.71	0.56 ± 0.67	0.67 ± 0.77	0.66 ± 0.68	0.65 ± 0.71	0.67 ± 0.70	0.68 ± 0.71	0.66 ± 0.70
Sleep disturbances	1.01 ± 0.46	0.91 ± 0.47	0.97 ± 0.49	0.91 ± 0.44	0.98 ± 0.46^∗^	0.86 ± 0.46	1.00 ± 0.47	0.93 ± 0.47	0.96 ± 0.49	0.93 ± 0.46	1.05 ± 0.45^∗^	0.90 ± 0.47
Daytime dysfunction	0.93 ± 0.78	1.04 ± 0.76	1.00 ± 0.74	1.02 ± 0.79	1.03 ± 0.76	0.95 ± 0.78	0.87 ± 0.76	1.04 ± 0.77	0.91 ± 0.78	1.05 ± 0.76	1.02 ± 0.77	1.00 ± 0.76
Habitual sleep efficiency	0.11 ± 0.46	0.05 ± 0.36	0.12 ± 0.49^∗^	0.03 ± 0.28	0.06 ± 0.34	0.09 ± 0.50	0.12 ± 0.51	0.06 ± 0.36	0.06 ± 0.39	0.07 ± 0.39	0.05 ± 0.27	0.08 ± 0.43
Sleep duration	1.30 ± 0.80	1.19 ± 0.77	1.27 ± 0.81	1.18 ± 0.75	1.26 ± 0.77	1.12 ± 0.79	1.12 ± 0.81	1.24 ± 0.77	1.26 ± 0.84	1.20 ± 0.75	1.32 ± 0.76	1.18 ± 0.78
Use of sleep medications	0.12 ± 0.51	0.08 ± 0.44	0.11 ± 0.51	0.08 ± 0.42	0.11 ± 0.49	0.06 ± 0.39	0.12 ± 0.51	0.09 ± 0.45	0.11 ± 0.51	0.09 ± 0.44	0.14 ± 0.55	0.08 ± 0.43

Data are presented as the mean ± SD. Comparison of sleep quality components between subjects with and without metabolic risk factors was performed using *t*-test. ^∗^*P* < 0.05.

**Table 4:** Crude and adjusted risk of metabolic syndrome for each increase score of sleep quality components in workers and employees.

	Workers		Employees	
	Crude risk	Adjusted risk^†^	Crude risk	Adjusted risk^†^
Poor sleep quality	2.76 (1.32-5.74)^∗∗^	3.04 (1.27-7.31)^∗^	1.27 (0.74-2.15)	1.48 (0.79-2.76)
Subjective sleep quality	1.89 (1.15-3.09)^∗^	2.29 (1.22-4.28)^∗∗^	0.92 (0.61-1.38)	1.01 (0.63-1.60)
Sleep latency	1.61 (1.02-2.55)^∗^	2.42 (1.31-4.49)^∗∗^	1.05 (0.74-1.49)	1.08 (0.72-1.62)
Sleep disturbances	2.59 (1.32-5.08)^∗∗^	2.43 (1.13-5.19)^∗^	1.54 (0.89-2.65)	1.19 (0.65-2.17)
Daytime dysfunction	1.66 (1.06-2.60)^∗^	2.06 (1.20-3.53)^∗∗^	0.81 (0.58-1.14)	0.80 (0.54-1.18)
Habitual sleep efficiency	0.64 (0.20-2.01)	0.68 (0.19-2.33)	1.36 (0.76-2.43)	1.74 (0.88-3.41)
Sleep duration	1.11 (0.74-1.68)	1.19 (0.69-2.06)	1.20 (0.87-1.66)	1.21 (0.84-1.76)
Using sleep medications	0.98 (0.51-1.88)	1.13 (0.54-2.36)	1.16 (0.70-1.93)	0.82 (0.43-1.55)

Analysis was performed using logistic regression with metabolic syndrome as a dependent factor. ^†^Adjusted for age, gender, BMI, shift work, and education. ^∗^*P* < 0.05 and ^∗∗^*P* < 0.01.

## Data Availability

The data used and/or analysed during the current study are available from the corresponding author on reasonable request.
